# Myocardin-Related Transcription Factor A (MRTF-A) Regulates the Balance between Adipogenesis and Osteogenesis of Human Adipose Stem Cells

**DOI:** 10.1155/2020/8853541

**Published:** 2020-09-22

**Authors:** Laura Hyväri, Sari Vanhatupa, Heidi T. Halonen, Minna Kääriäinen, Susanna Miettinen

**Affiliations:** ^1^Adult Stem Cell Group, Faculty of Medicine and Health Technology, Tampere University, Tampere, Finland; ^2^Research, Development and Innovation Centre, Tampere University Hospital, Tampere, Finland; ^3^Computational Biophysics and Imaging Group, Faculty of Medicine and Health Technology, Tampere University, Tampere, Finland; ^4^Department of Plastic and Reconstructive Surgery, Tampere University Hospital, Tampere, Finland

## Abstract

Previous studies have demonstrated that myocardin-related transcription factor A (MRTF-A) generates a link between the dynamics of the actin cytoskeleton and gene expression with its coregulator, serum response factor (SRF). MRTF-A has also been suggested as a regulator of stem cell differentiation. However, the role of MRTF-A in human mesenchymal stem cell differentiation remains understudied. We aimed to elucidate whether MRTF-A is a potential regulator of human adipose stem cell (hASC) differentiation towards adipogenic and osteogenic lineages. To study the role of MRTF-A activity in the differentiation process, hASCs were cultured in adipogenic and osteogenic media supplemented with inhibitor molecules CCG-1423 or CCG-100602 that have been shown to block the expression of MRTF-A/SRF-activated genes. Our results of image-based quantification of Oil Red O stained lipid droplets and perilipin 1 staining denote that MRTF-A inhibition enhanced the adipogenic differentiation. On the contrary, MRTF-A inhibition led to diminished activity of an early osteogenic marker alkaline phosphatase, and export of extracellular matrix (ECM) proteins collagen type I and osteopontin. Also, quantitative Alizarin Red staining representing ECM mineralization was significantly decreased under MRTF-A inhibition. Image-based analysis of Phalloidin staining revealed that MRTF-A inhibition reduced the F-actin formation and parallel orientation of the actin filaments. Additionally, MRTF-A inhibition affected the protein amounts of *α*-smooth muscle actin (*α*-SMA), myosin light chain (MLC), and phosphorylated MLC suggesting that MRTF-A would regulate differentiation through SRF activity. Our results strongly indicate that MRTF-A is an important regulator of the balance between osteogenesis and adipogenesis of hASCs through its role in mediating the cytoskeletal dynamics. These results provide MRTF-A as a new interesting target for guiding the stem cell differentiation in tissue engineering applications for regenerative medicine.

## 1. Introduction

The actin cytoskeleton of a cell is continuously modified to allow dynamic cell functions such as stem cell differentiation [1, 2]. Actin dynamics are accomplished by continuous actin turnover and treadmilling regulated by Rho GTPase RhoA-Rho-associated coiled-coil kinase (Rho-ROCK) pathway [3, 4]. RhoA-ROCK pathway has also been reported to regulate the fate decision of mesenchymal stem cells (MSCs) [1, 2, 5]. However, the role of ROCK downstream effector myocardin-related transcription factor A (MRTF-A) in the regulation of MSC differentiation remains less studied. MRTF-A, also known as megakaryocyte acute leukemia protein (MAL) and megakaryoblastic leukemia (MKL1), belongs to the myocardin family [6, 7] and is found in numerous embryonic and adult tissues [8]. MRTF-A generates a unique link between actin dynamics and gene expression because the activity of MRTF-A is controlled by the balance between monomeric G-actin and the polymerized filamentous F-actin. At low actin polymerization state, MRTF-A is inactive as it is reversibly bound to cytoplasmic or nuclear G-actin through N-terminal RPEL repeats. On the other hand, when actin filament assembly is stimulated by Rho-ROCK activity, MRTF-A is released from its repressive complex with G-actin [7, 9, 10]. In the nucleus, an active MRTF-A works as a transcription coactivator of serum response factor (SRF) to activate transcription of contractile and cytoskeletal genes including alpha-, beta-, and gamma actins, integrin *β*1, vinculin; cofilin 1; talin 1; myosin heavy chains; and myosin light chain 9 [6–8, 11].

Recently, studies have linked MRTF-A to regulation of adipogenesis through its ability to respond to actin dynamics [12]. Adipogenic differentiation towards white adipose tissue (WAT) has been reported to involve cytoskeletal changes towards rounder cell morphology, which in mature adipocytes allows optimal lipid storage [13, 14]. Adipogenesis is regulated through sequential activation of transcription factors, of which peroxisome proliferator-activated receptor gamma (PPAR*γ*) is considered the key adipocyte-specific master switch [13–16]. Interestingly, PPAR*γ* has been found to be one of the targets through which MRTF-A regulates adipogenesis [17, 18]. Nobusue and coworkers reported that depletion of MRTF-A with RNAi method enhanced PPAR*γ* in murine cells [12]. To support this finding, McDonald and coworkers observed that MRTF-A and SRF were downregulated during adipogenesis of murine embryo fibroblasts, and reciprocally, cells genetically manipulated to express MRTF-A and SRF displayed hindered adipogenesis [19]. Similarly, in a study of Mikkelsen and coworkers, SRF overexpression inhibited adipogenesis, whereas RNAi-mediated knockdown of SRF enhanced adipogenesis in mouse preadipocytes [20]. In addition to regulation of WAT, MRTF-A has been reported to regulate the browning of WAT containing mitochondria and uncoupling protein 1- (UCP1-) rich cells found in brown and beige adipose tissue [17, 19].

Adipogenesis and osteogenesis have been found mutually exclusive in MSC differentiation [2, 5, 15], Thus, we hypothesized if MRTF-A would as well reciprocally guide the differentiation fate decision. The MSC osteogenesis is controlled by a master regulator runt-related transcription factor 2 (RUNX2), which regulates the osteoblast-specific gene expression of alkaline phosphatase (ALP), osteopontin (OPN), and osteocalcin (OCN) [15, 21]. The actin cytoskeleton is also modified during osteoblast formation: angular shape, well-defined stress fibers, but also increased actin polymerization have been reported [1, 22, 23]. To date, the existing literature addressing MRTF-A in osteogenic differentiation is sparse. Bian and coworkers implicated the regulatory effect of MRTF-A on osteogenic differentiation potential in rodent MSCs [24]. In their study, MRTF-A knockout mice had inferior bone development compared with wild type, and bone marrow mesenchymal stem cells (BMSCs) isolated from the knockout mice had decreased osteogenesis *in vitro* [24]. Supporting these findings, also, SRF-deficient mice have been reported to have reduced bone mineral density and bone formation rate [25].

Our study aimed to investigate the role of MRTF-A coregulator in guiding the differentiation commitment of human adipose stem cells (hASCs). Human ASCs are multipotential MSCs with the capacity to give rise to mesenchymal tissues such as fat, bone, and cartilage and have immunomodulatory properties making them suitable to be used in regenerative medicine [26]. Our approach to study the role of MRTF-A in the regulation of differentiation fate was to use two molecular inhibitors described by Evelyn and coworkers targeted to MRTF-A/SRF-mediated gene transcription, a first-generation inhibitor CCG-1423 [27] and a second-generation analog CCG-100602 [28]. CCG-100602 was designed and synthesized to improve the potency and attenuate the cytotoxicity of the lead compound by molecular modifications to the chemical structure of CCG-1423 [28–30]. The molecular target of CCG-1423 has been proposed to be the N-terminal basic domain of MRTF-A which acts as a functional nuclear localization signal (NLS) [31]. In contrast, the biological activity of the related compound CCG-100602 remains unidentified. We cultured hASCs in basic culture medium (BM) and differentiated the cells using adipogenic and osteogenic culture media, AM and OM, respectively, with or without inhibitor supplementation. Adipogenesis and osteogenesis were studied using analyses of early and late markers of differentiation, immunocytochemical staining of both intracellular and extracellular matrix (ECM) proteins, and image-based analysis methods. In addition, the F-actin formation, orientation of actin filaments, and actin-related proteins were studied to evaluate the role of MRTF-A in mediating the cytoskeletal responses during differentiation. To the best of our knowledge, this is the first study elucidating the role of MRTF-A in the regulation of the balance between hASC adipogenic and osteogenic differentiation courses. Since hASCs have been increasingly used in the clinical setting, the detailed understanding of their molecular mechanisms is of great value.

## 2. Materials and Methods

### 2.1. hASC Isolation and Culture

The hASCs used in the study were isolated from adipose tissue samples of six female donors aged 40–63 (Md = 51) with their written informed consent in accordance with the Regional Ethics Committee of the Expert Responsibility area of Tampere University Hospital, Tampere, Finland (ethical approval R15161). More detailed donor information is given in Table S1. The isolation protocol has been described previously [32, 33]. Briefly, the adipose tissue was digested mechanically and enzymatically (Collagenase type I; Thermo Fisher Scientific; Waltham, MA, USA), centrifuged, and filtrated to separate the stem cells.

The isolated hASCs were cultured adhering to a Nunclon Delta surface polystyrene culture flask (Thermo Fisher Scientific). Human ASCs were expanded and cultured in basic culture medium, designated as BM, containing 5% human serum (GE Healthcare; Chicago, IL, USA, or BioWest; Nuaillé, France) and 1% antibiotics (Penicillin/Streptomycin; Lonza, Basel, Switzerland) in Minimum Essential Medium *α*, no nucleosides (MEM *α*), or Dulbecco's Modified Eagle Medium/Ham's Nutrient Mixture F-12, no glutamine (DMEM/F-12) (both media from Thermo Fisher Scientific). 1% GlutaMax (Thermo Fisher Scientific) was added when using DMEM/F-12. The cells were passaged when reaching 70–80% confluency and detached using TrypLE Select (Thermo Fisher Scientific).

### 2.2. Flow Cytometry Analysis of Immunophenotype

The hASCs used in the study were characterized by flow cytometry (FACSAria; BD Biosciences, Erembodegem, Belgium) at passage 1 to evaluate their immunophenotype. Human ASCs (10 000 cells/sample) were single stained with monoclonal antibodies: CD14-PE-Cy7, CD19-PE-Cy7, CD45RO-APC, CD73-PE, CD90-APC (aforementioned antibodies from BD Biosciences, Franklin Lakes, NJ, USA), CD11a-APC, CD105-PE (R&D Systems Inc., Minneapolis, MN, USA), CD34-APC, and HLA-DR-PE (Immunotools GmbH, Friesoythe, Germany). Fluorescence level greater than 99% was considered positive.

### 2.3. Differentiation Media and Inhibitors

Human ASCs were induced with differentiation culture media and the MRTF-A inhibitors 24 h after plating the cells for experiments. Osteogenic differentiation was accomplished with osteogenic medium (OM) consisting of BM supplemented with 200 *μ*M L-ascorbic acid 2-phosphate, 10 mM *β*-glycerophosphate, and 5 nM dexamethasone (DEX) (reagents from Sigma-Aldrich, Saint Louis, MO, USA). For adipogenic differentiation, hASCs were cultured in adipogenic medium (AM) containing BM supplemented with 1 *μ*M DEX, 17 *μ*M pantothenate, 33 *μ*M biotin (from Sigma-Aldrich), and 100 nM insulin (Thermo Fisher Scientific). 0.25 mM 3-isobutyl-1-methylxanthine (IBMX; Sigma-Aldrich) was added to AM once at the beginning of differentiation. MRTF-A inhibition was carried out by supplementing BM, OM, and AM with one of two inhibitor molecules targeted to MRTF-A/SRF signaling. We used a first-generation inhibitor CCG-1423 (Selleck Chemicals; Houston, TX, USA) and a second-generation analog CCG-100602 (Cayman Chemical Company; Ann Arbor, MI, USA). BM, OM, and AM without inhibitors were used as controls. Media and inhibitors were changed twice a week during the experiments.

### 2.4. Cell Viability

The inhibitor effect on the viability of hASCs was analyzed with LIVE/DEAD™ Viability/Cytotoxicity Kit for mammalian cells (Invitrogen™; Thermo Fisher Scientific). The cells were seeded on CellBIND culture plates (Corning) in the density of 260 cells/cm^2^ and cultured 7 days in BM, OM, or AM conditions and the media supplemented with 3 *μ*M, 8 *μ*M, 15 *μ*M, or 30 *μ*M CCG-100602 or 15 *μ*M, 20 *μ*M, 25 *μ*M, or 30 *μ*M CCG-1423. After the 7-day culture, staining was done as described previously [34], but using a 30 min incubation time for the staining solution. Briefly, the cells were washed with Dulbecco's Phosphate-Buffered Saline (DPBS; Lonza) and stained with a working solution of 0.5 *μ*M calcein-AM and 0.25 *μ*M ethidium homodimer-1 (EthD-1) in DPBS for 30 min at room temperature. After staining, fresh DPBS was changed to the wells, and the viable green-stained (calcein-AM) and dead red-stained (EthD-1) cells were immediately imaged.

### 2.5. Proliferation and ALP Activity

The proliferation of hASCs was analyzed using CyQUANT™ assay (Invitrogen™; Thermo Fisher Scientific). The hASCs were seeded on CellBIND culture plates (Corning) in the density of 260 cells/cm^2^ and cultured for 7 days or 14 days in BM, OM, or AM conditions supplemented with 15, 20, or 25 *μ*M CCG-1423 inhibitor or 10 or 12 *μ*M CCG-100602 inhibitor. BM, OM, and AM without inhibitors were used as controls. The hASCs were lysed into 0.1% Triton X-100 buffer (Sigma-Aldrich). After a freeze-thaw cycle, the proliferation of hASCs was studied with CyQUANT GR-Dye in lysis buffer, and the fluorescence was measured with a microplate reader at 480/520 nm (Wallac Victor 1420 Multilabel Counter; Perkin Elmer, Waltham, MA, USA).

The activity of ALP, an early marker of osteogenic differentiation, was studied from the same samples as proliferation, but only the samples cultured in BM and OM conditions were studied. The lysed samples were analyzed with a colorimetric assay, as described before [35]. Briefly, the samples were incubated 15 min 37°C in a working solution containing 1 : 1 10.8 *μ*M phosphatase substrate and 1.5 M alkaline buffer solution. After incubation, the reaction was halted using 1.0 M sodium hydroxide (all reagents from Sigma-Aldrich). Absorbances were measured at 405 nm.

### 2.6. Alizarin Red Staining and Quantification of Mineralization

Late osteogenic differentiation was evaluated by assessing the calcium accumulation with Alizarin Red (AR) staining. The hASCs were seeded 260 cells/cm^2^ on CellBIND plates (Corning) and cultured 21 days in BM or OM conditions with or without CCG-1423 and CCG-100602 inhibitors. At day 21, the cells were fixed with 70% ethanol and stained with 2% Alizarin Red S solution (pH 4.1–4.3; Sigma-Aldrich) for 10 min. After staining, wells were washed with deionized water and 70% ethanol and dried. Samples were photographed with Olympus OM-D E-M5 Mark II camera with M. Zuiko 60 mm macro lens (Olympus; Tokyo, Japan). Quantification of the staining was done by eluting the dye into 100 mM cetylpyridinium chloride (Sigma-Aldrich) for 3.5 h and measuring the absorbances at 544 nm.

### 2.7. Oil Red O Staining

Adipogenesis was studied by assessing the accumulation of lipid droplets with Oil Red O (ORO) staining. The hASCs were seeded 260 cells/cm^2^ on CellBIND plates (Corning) and cultured for 21 days in BM or AM conditions. The media were supplemented with 15 or 20 *μ*M CCG-1423 or 10 or 12 *μ*M CCG-100602 inhibitors. BM and AM without inhibitors were used as controls. At day 21, the cells were fixed with 4% paraformaldehyde (PFA; Sigma-Aldrich), rinsed with deionized water, and pretreated with 60% isopropanol (2-propanol, Merck, Darmstadt, Germany). The cells were stained with 0.2% ORO staining solution for 15 min, counterstained 5 min with 1 : 2000 4′,6-diamidino-2-phenylindole (DAPI; Sigma-Aldrich) in deionized water, and washed several times.

### 2.8. Immunocytochemical and Phalloidin Staining

The hASCs were seeded 1000 cells/cm^2^ into chambered polymer coverslips (ibiTreat *μ*-slide 8-well chamber; Ibidi GmbH, Gräfelfing, Germany) and cultured in BM, OM, or AM supplemented with 20 *μ*M CCG-1423 or 12 *μ*M CCG-100602. The culture period was 7 days for Phalloidin staining, 14 days for immunocytochemical staining (ICC) of collagen type I (COL-1), and 21 days for ICC staining of osteopontin (OPN) and perilipin 1 (Plin1). After culture, hASCs were washed with PBS (Lonza), fixed 15 min with 0.2% triton X-100 in PFA (Sigma-Aldrich), and blocked with 1% Bovine serum albumin (BSA; Sigma-Aldrich) in PBS. The samples were incubated with primary antibodies, washed, and incubated with secondary antibodies supplemented with Phalloidin-TRITC. After washes, the samples were counterstained with DAPI. Detailed reagent information is given in Table 1. Negative controls are presented in Figure S2.

### 2.9. Fluorescence Imaging

LIVE/DEAD-, ORO-, ICC-, and Phalloidin-stained hASC samples were imaged under an inverted microscope Olympus IX51 (Olympus) equipped with fluorescence unit and camera (DP30BW). Fluorescence images were taken using Alexa 488, Alexa 546, and DAPI filters, and 4, 10, 20, or 40x objectives. The ORO-, ICC- (COL-1, OPN, and Plin1), and Phalloidin-stained samples for quantification of mean intensity were imaged keeping the exposure times constant between the samples of different culture conditions. On the contrary, the LIVE/DEAD- and Phalloidin-stained samples for image-based analysis of actin orientation were imaged by adjusting optimal exposure times for each image to ensure visibility of the cells and the actin filaments, respectively. The image processing was done with Adobe Photoshop CC (Adobe; San Jose, CA, USA) or Fiji [36].

### 2.10. Image-Based Quantification of Lipid Droplet Area

Lipid droplet formation was quantified based on image analysis of ORO-stained samples with a custom analysis pipeline designed for CellProfiler (version 2.0.0, 64-bit Windows) [37]. The same protocol was used as described previously [5], but 0.1 and 1.0 were set as lower and upper bounds for lipid area segmentation, and 15 pixels was set as a threshold for exclusion of lipid droplet areas smaller than 5 *μ*m in diameter.

### 2.11. Image-Based Analysis of F-Actin Intensity and Orientation

In an effort to analyze the formation of actin filaments, the Phalloidin- and DAPI-stained 40x fluorescence images of hASCs were analyzed with Fiji [36]. Briefly, the mean intensity of Phalloidin in each condition was measured, and the nuclei count of corresponding images was analyzed for normalization of the intensities.

For the analysis of actin orientation, 20x fluorescence images of Phalloidin-stained hASCs were analyzed with CytoSpectre 1.2 spectral analysis tool [38]. In brief, the images were analyzed with default settings, but specifying image magnification (20x) and camera pixel size (6.45 *μ*m). The software was used to calculate the circular variance describing the isotropy of orientation distribution in the image field. Circular variance is bounded in the interval [0, 1], where a value closer to zero signifies distribution along the same direction (anisotropy), and a value closer to one designates spread distribution (isotropy) [38, 39].

### 2.12. Western Blotting and Immunodetection

Western blotting was performed to detect the protein expression of inhibitor-treated hASCs. The cells were seeded 5000 cells/cm^2^ in CellBIND 6-well plate (Corning) and cultured 7 days in BM, OM, or AM supplemented with 20 *μ*M CCG-1423 or 12 *μ*M CCG-100602. Samples were washed with PBS (Lonza), lysed with 2x LAEMMLI sample buffer (Bio-Rad; Hercules, CA, USA), and separated with sodium dodecyl sulfate polyacrylamide gel electrophoresis. After separation, the proteins were transferred onto 0.2 *μ*m polyvinylidene fluoride (PVDF) membrane using Trans-Blot Turbo Ready-to-assemble transfer Kit (Bio-Rad). The PVDF membranes were blocked with 5% nonfat milk powder in Tris-buffered saline (TBS) supplemented with 0.05% Tween 20 (Sigma-Aldrich). Membranes were incubated with primary antibodies followed by washing steps (0.5%, 0.1%, and 0.05% Tween 20 in TBS) and secondary antibody incubation (antibodies, dilutions, and incubation times are presented in Table 2). After similar washing steps, proteins of interest were detected with chemiluminescence (ECL Prime Western Blotting Detection Reagent; GE Healthcare, Little Chalfont, UK), and the membranes were imaged with Chemi Doc MP System (Bio-Rad). Semiquantitative analysis of immunoblotted protein amounts was performed with Image J [40] to show the protein levels of MRTF-A, *α*-SMA, pMLC, and MLC normalized with *β*-actin representing the cell amount in different culture conditions.

### 2.13. Statistical Analysis

Statistical significances were analyzed separately within each culture media (BM, OM, or AM) by comparing the inhibited conditions to the control condition without the inhibitors. MRTF-A inhibitor effects on cell proliferation (CyQUANT, 4 donors), ALP activity (4 donors), quantitative Alizarin Red staining (3 donors), and image-based analysis of actin (2 donors) and ORO (4 donors) were evaluated. All quantitative results are presented as mean and standard deviation (SD). Nonparametric Kruskal-Wallis and Mann-Whitney tests were performed using GraphPad Prism version 5.02 (GraphPad Software; La Jolla, CA, USA), followed by Bonferroni post hoc test. *N* values represent the number of parallel samples or images analyzed. The differences with *p* ≤ 0.05 were considered significant.

## 3. Results

### 3.1. hASC Characterization

The cells were identified as mesenchymal based on their plastic adherence, differentiation potential towards adipogenic and osteogenic lineages, and the surface marker expression pattern conveying the criteria given by the International Society for Cellular Therapy [41]. The expression of CD73, CD90, and CD105 was positive (≥95% cells), and there was a lack of expression of CD11a, CD14, CD19, CD45, and HLA-DR (≤2% positive cells). The expression of hematopoietic marker CD34 was the modest, but previous studies have linked this elevation to the early passage of ASCs that declines when the cells are passaged [14, 42, 43]. Detailed surface marker expressions, their standard deviations, and fluorophore information are given in Table S2.

### 3.2. Viability and Proliferation

**Figure 1:**
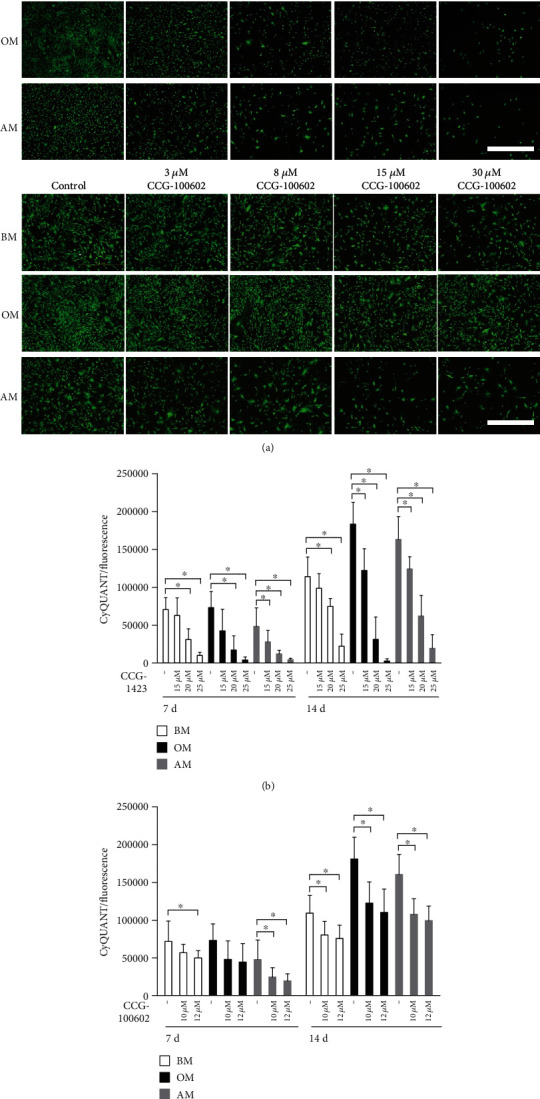
Effect of MRTF-A inhibition on viability and proliferation of hASCs. (a) Representative fluorescence images of LIVE/DEAD-stained hASCs. hASCs were cultured 7 d in BM, OM, or AM supplemented with 15, 20, 25, or 30 *μ*M CCG-1423 or 3, 8, 15, or 30 *μ*M CCG-100602 inhibitor, after which LIVE/DEAD analysis was performed. Green dye represents living cells (Alexa 488 filter), and a negligible number of dead cells are stained with red dye (Alexa 546). Scale bar 1.0 mm, same scale in every image. (b, c) Proliferation of hASCs at 7 and 14 d was analyzed with the CyQUANT method. The hASCs were cultured in BM, OM, or AM supplemented with 15, 20, or 25 *μ*M CCG-1423 inhibitor (b) or 10 or 12 *μ*M CCG-100602 inhibitor (c). *N* = 12, independent biological replicates from 4 donors. 5% significance level was used in the statistical analysis, and the comparisons were made within a culture condition by comparing the inhibitor concentrations with the untreated medium control. BM: basic medium; OM: osteogenic medium; AM: adipogenic medium.

We first examined the role of MRTF-A in hASC adhesion and viability by using two inhibitor molecules (CCG-1423 and CCG-100602) in BM, OM, and AM cultures. A selection of concentrations of both inhibitors was used to find optimal concentrations for cell viability. Based on the representative LIVE/DEAD-stained images of adherent hASCs in Figure 1(a), there were viable green-stained cells, but no dead cells, in all culture conditions. The number of adherent cells decreased as a response to increasing inhibitor amount. With CCG-100602, the effect was also dependent on the culture media because the OM condition supported the viability over BM and AM conditions.

Cell proliferation, analyzed with CyQUANT assay, aligned with the viability results. Figures 1(b) and 1(c) show that the inhibitors had a dose-dependent effect on the cellular nucleic acid bound fluorescent GR-Dye denoting cell number. The inhibitor effect accumulated over time, and more significant reductions in cell amounts were seen after 14 days of culture. The hASCs in AM were more sensitive to inhibition, and lower inhibitor concentrations led to significantly reduced cell amount already at day 7.

### 3.3. Adipogenic Differentiation

**Figure 2:**
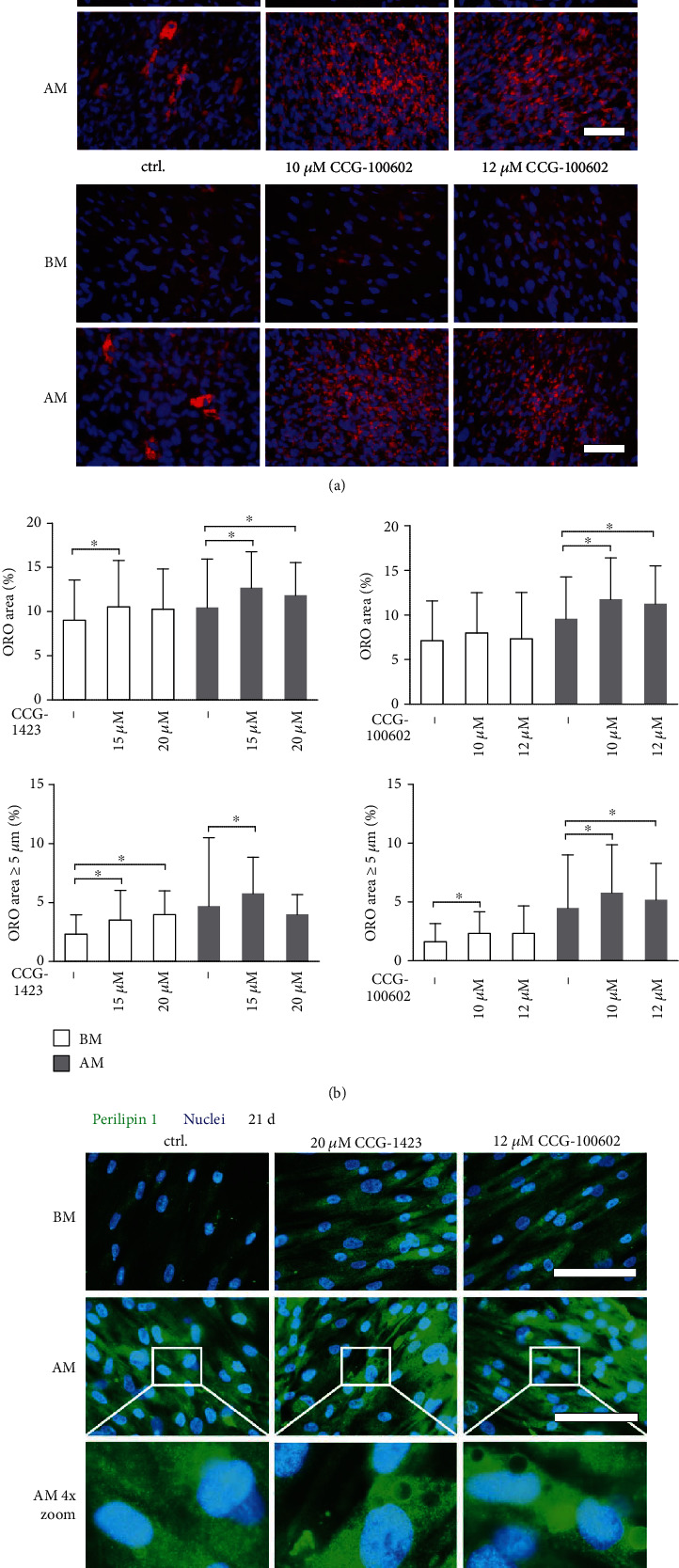
Adipogenesis of hASCs treated with MRTF-A inhibitors at 21 d. hASCs were cultured 21 d in BM or AM conditions supplemented with 15 or 20 *μ*M CCG-1423 or 10 or 12 *μ*M CCG-100602 inhibitors and stained with ORO method. (a) Representative fluorescence images of hASCs stained with ORO for intracellular lipid accumulation followed by nuclei staining with DAPI. Fluorescence images were taken with Alexa 546 for ORO (red) and DAPI (blue) filters, and 20x magnification. Scale bars 100 *μ*m. (b) Lipid droplet areas were quantified from the ORO-stained fluorescence images with a custom analysis pipeline designed for CellProfiler. The graphs present the total ORO-stained area or lipid droplet clusters with a diameter of over 5 *μ*m as percentages of the total image area. *N* = 36 for BM +20 *μ*M CCG-1423 and AM +20 *μ*M CCG-1423, other conditions *N* = 59 − 63 images (15 independent biological replicates from 4 donors). (c) Representative Plin1- and DAPI-stained samples of MTRF-A inhibitor-treated hASCs at 21 d imaged with a fluorescence microscope using Alexa 488 for Plin1 (green) and DAPI (blue) filters, and 40x magnification. The lowest row represents 4x digital zoom of the white rectangles in AM images. Scale bars 100 *μ*m. BM: basic medium; AM: adipogenic medium; ORO: Oil Red O.

We next investigated the significance of MRTF-A activity on the adipogenic potential of hASCs using ORO staining of cytoplasmic neutral lipids after 21 days of culture in BM or AM conditions supplemented with 15 or 20 *μ*M CCG-1423 or 10 or 12 *μ*M CCG-100602. The representative ORO- and DAPI-stained fluorescence images are shown in Figure 2(a). The lipid droplet accumulation was also quantified by analyzing the total ORO-stained area, as well as the area of lipid droplet clusters exceeding 5 *μ*m in diameter to demonstrate the adipogenic maturation (Figure 2(b)). BM condition contained small red-stained lipid droplets, and both inhibitors significantly increased the size of the individual droplets. Noticeable adipogenic differentiation with large matured droplets was obtained in the AM condition, but the enlarged, clustered lipid droplets were quite scarce in AM control. MRTF-A inhibition significantly enhanced both the total area of lipid droplets and the proportion of large droplets in AM condition. However, despite their abundance, the MRTF-A inhibitor-induced lipid droplets were not as clustered as in AM control but rather more separated. Interestingly, 15 *μ*M CCG-1423 and 10 *μ*M CCG-100602 were found more optimal concentrations in supporting the hASC maturation.

Adipogenic differentiation was further assessed with ICC staining of adipogenic marker Plin1 (Figure 2(c)). The inhibitor treatment enhanced Plin1 production of the hASCs in BM condition. Plin1 staining was stronger in AM conditions, and the staining intensity was relatively the same in control AM and with inhibition. The zoom images revealed large lipid droplets covered with Plin1 in the AM supplemented with MRTF-A inhibitors. However, these structures were absent in the AM control condition. In addition, quantitative real-time PCR (qRT-PCR) analysis was done to study the effect of MRTF-A inhibition on a genetic level using one donor cell line (Table S3 and Figure S1). We found that the MRTF-A inhibitors led to enhanced or unaffected human adipocyte fatty acid-binding protein (*AP2*) expression, but both inhibitors had a downregulating effect on leptin (*LEP*) in AM condition.

### 3.4. Osteogenic Differentiation

**Figure 3:**
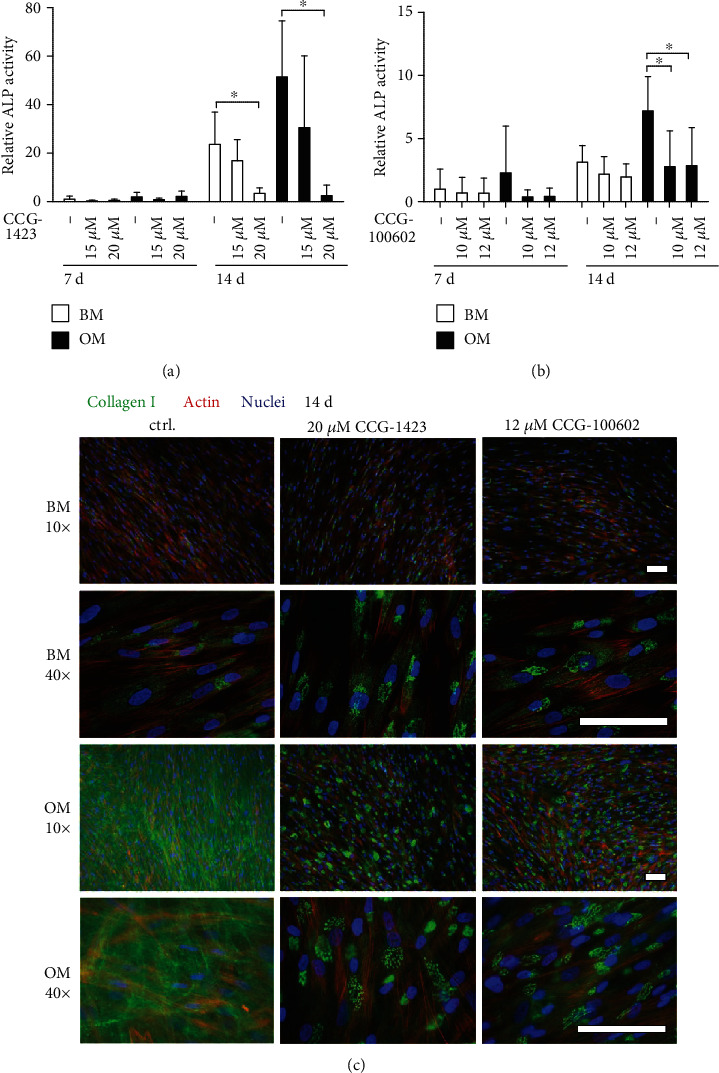
Early osteogenesis of MRTF-A inhibitor-treated hASCs. The hASCs were cultured in BM or OM supplemented with CCG-1423 (a) or CCG-100602 (b) inhibitors in addition to medium controls. ALP activity was analyzed with ALP assay at 7 d and 14 d. The ALP absorbance values were normalized with corresponding CyQUANT results, and the results are presented relative to the 7 d BM sample. *N* = 12, independent biological replicates from 4 donors. Significance level 5%. (c) Representative images of COL-1 (Alexa 488, green)-, actin (Alexa 546, red)-, and nuclei (DAPI, blue)-stained hASCs at 14 d, after culture with 20 *μ*M CCG-1423 or 12 *μ*M CCG-100602. Images with 10x and 40x magnifications are provided to give an overall view and a more detailed view of intracellular localization of COL-1. Scale bars 100 *μ*m. BM: basic medium; OM: osteogenic medium.

We evaluated the early osteogenic potential of MRTF-A inhibitor-treated hASCs by measuring the activity of ALP, at day 7 and 14 time points in BM or OM conditions. Figures 3(a) and 3(b) show that the ALP activity remained relatively low at day 7, and its activity rose within the culture period. The ALP activity was elevated in OM control condition at day 14, but decreased dose-dependently with MRTF-A inhibition in both BM and OM conditions. The activity was significantly reduced in OM with only 25 *μ*M CCG-1423, whereas the next-generation inhibitor CCG-100602 reduced ALP significantly with both concentrations. Additionally, the gene expression of an early osteogenic marker gene runt-related transcription factor 2a (*RUNX2A*) was decreased with CCG-1423 when analyzed with qRT-PCR (Table S3 and Figure S1).

The osteogenesis-related formation of secreted ECM proteins was analyzed by COL-1 staining at day 14. In BM, although the level of staining remained modest, inhibition of MRTF-A slightly increased COL-1 synthesis and processing in the endoplasmic reticulum (ER) and Golgi network (Figure 3(c)). In OM, however, COL-1 staining was considerably stronger, and the protein was secreted into ECM to form fibrous structures in the control condition. COL-1 production was lower in OM conditions with MRTF-A inhibitors, and the protein was sequestrated to the intracellular membranes.

**Figure 4:**
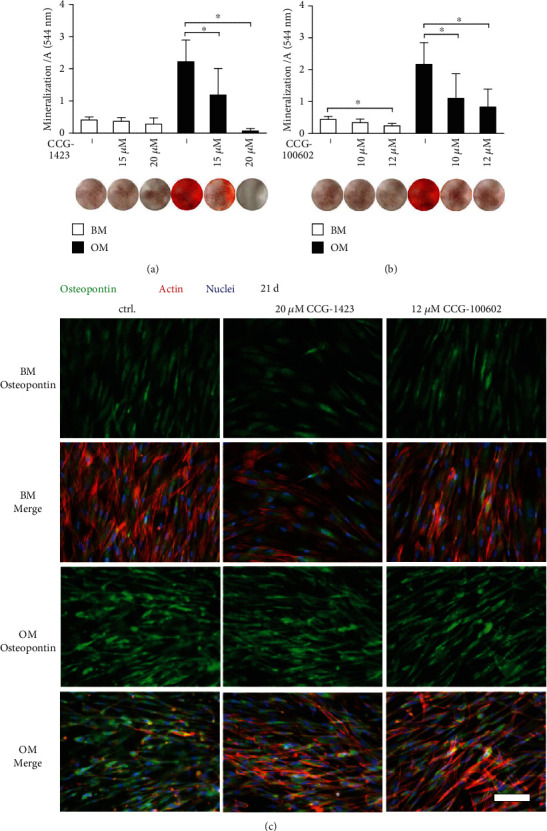
Late osteogenesis of MRTF-A inhibitor-treated hASCs. Matrix mineralization of hASCs was analyzed with AR staining after 21 d of culture with CCG-1423 (a) or CCG-100602 (b) inhibitors. Quantitative results of AR staining are presented as graphs, and corresponding representative images of the stained wells (area 1.9 cm^2^) are displayed below the columns, bright red dye represents mineral. *N* = 9, independent biological replicates from 3 donors. Significance level 5%. (c) Representative OPN-stained samples of MTRF-A inhibitor-treated hASCs at 21 d. The cells were imaged with a fluorescence microscope using Alexa 488 for OPN (green), Alexa 546 for actin (red), and DAPI (blue) filters, and 20x magnification. Scale bar 100 *μ*m, same scale in every image. BM: basic medium; OM: osteogenic medium.

The ability of the hASCs to differentiate further towards osteoblasts was evaluated by AR staining of calcium deposits at day 21 for the assessment of ECM mineralization (Figures 4(a) and 4(b)). AR staining was low in BM conditions, as hypothesized, and the polystyrene control for AR staining was clear (data not shown). In OM control condition, the hASCs showed calcium accumulation as represented by the strong red staining of the samples. Inhibition of MRTF-A signaling by CCG-1423 or CCG100602 resulted in a significant dose-dependent reduction of mineral formation in the OM condition. Late osteogenesis was also analyzed by ICC staining of OPN. As displayed in Figure 4(c), the hASCs produced OPN independent of the culture condition. However, cells in OM without inhibitors were producing and secreting OPN to the ECM unlike the other culture conditions. Interestingly, MRTF-A inhibition confined OPN in the intracellular space.

### 3.5. Actin-Related Proteins and Cytoskeleton

**Figure 5:**
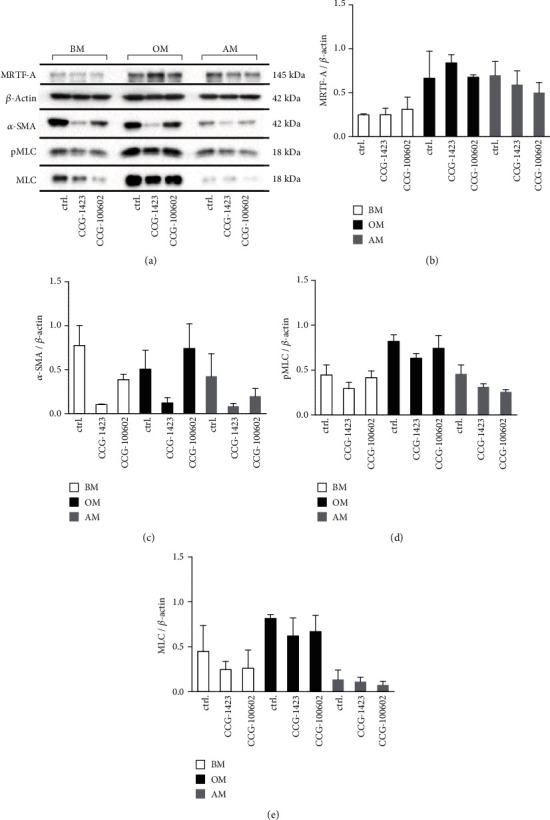
Intracellular protein levels of MRTF-A, *β*-actin, *α*-SMA, pMLC, and MLC as a response to MRTF-A inhibition. hASCs were cultured 7 d in BM, OM, or AM media supplemented with 20 *μ*M CCG-1423 or 12 *μ*M CCG-100602. (a) Representative WB results of immunoblotted MRTF-A, *β*-actin, *α*-SMA, pMLC, and MLC. Semiquantitative results of MRTF-A (b), *α*-SMA (c), pMLC (d), and MLC (e) normalized with *β*-actin. MRTF-A and *α*-SMA: *N* = 2 independent experiments, 2 donors; pMLC and MLC: *N* = 4 independent experiments, 2 donors. BM: basic medium; OM: osteogenic medium; AM: adipogenic medium.

The role of MRTF-A in the synthesis of actin-related proteins was assessed by Western blotting and immunodetection of MRTF-A, *β*-actin, *α*-SMA, pMLC, and MLC at day 7 (Figure 5 and Figure S3). Based on the band size and intensity, the total protein amount of *β*-actin was relatively constant in the studied culture conditions, and the inhibitor effect on the protein level of MRTF-A varied in different culture media. However, MRTF-A inhibition predominantly decreased the amount of SRF-regulated proteins *α*-SMA, MLC, and the phosphorylated form pMLC, although the culture media supplements also played a role on protein expression. We found that both inhibitors reduced the *α*-SMA protein amount in BM and AM conditions, but in OM condition, only CCG-1423 reduced *α*-SMA. MLC and its phosphorylated form pMLC were induced in OM, and their protein levels were lower in BM and AM conditions.

**Figure 6:**
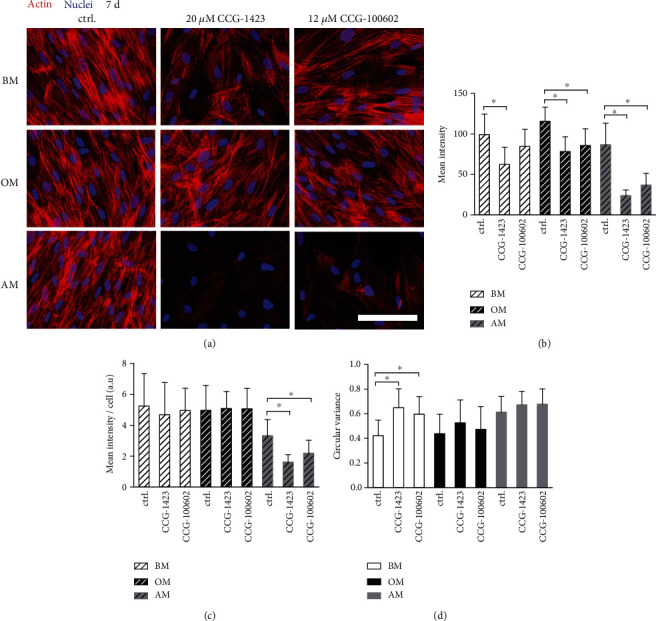
F-actin intensity and orientation of MRTF-A inhibitor-treated hASCs. hASCs were cultured 7 d in BM, OM, or AM media supplemented with 20 *μ*M CCG-1423 or 12 *μ*M CCG-100602. (a) Representative images of Phalloidin- and DAPI-stained hASCs imaged with Alexa 546 for actin (red) and DAPI for nuclei (blue) filters using 40x magnification and constant exposure time. Scale bar 100 *μ*m. Image-based analysis done with Fiji of mean Phalloidin intensity (b) and mean intensity normalized with nuclei number (c) of the samples described in (a). *N* = 18‐25 images, 4 independent biological replicates from 2 donors. Significance level 5%. (d) Image-based analysis of actin orientation done with CytoSpectre 1.2 spectral analysis tool. Phalloidin-stained hASCs were imaged with 20x magnification and optimally adjusted exposure times from the same biological replicates as above (representative images in Figure S4). *N* = 16‐27 images. Significance level 5%. BM: basic medium; OM: osteogenic medium; AM: adipogenic medium.

In order to study the cytoskeleton of MRTF-A inhibitor-treated hASCs in BM, OM, and AM conditions, the F-actin of the cells was stained with Phalloidin-TRITC (Figure 6(a)). The hASCs were fibroblastic and spindle-like in BM and OM control conditions. MRTF-A inhibition forced the cells to adopt more spread morphology with less coherently aligned cytoskeleton. Additionally, the inhibitor treatment led to decreased mean Phalloidin intensity in every culture media (Figure 6(b)). The inhibitor effect was the most prevalent in AM condition where the mean intensity values normalized with nuclei count of corresponding images were significantly reduced (Figure 6(c)). The circular variance, i.e., the isotropy of the actin filament orientations, was the lowest in BM and OM control conditions, meaning that the actin filaments were the most parallel-aligned (Figure 6(d), representative images in Figure S4). Based on quantitation, MRTF-A inhibition significantly increased the circular variance of the actin filaments in BM condition, and a similar trend was found in OM and AM conditions. The adipogenic culture medium itself resulted in less fibroblastic morphology compared with BM and OM.

## 4. Discussion

The dynamic behavior of the actin cytoskeleton is central in many cellular processes, including stem cell commitment into various differentiation lineages. Importantly, the changes in cell morphology have also been proposed to guide the differentiation. [1, 2] Actin dynamics are accomplished by actin turnover through the reversible polymerization of actin monomers into filaments. The ratio of G-actin and F-actin, in turn, regulates the activity of MRTF-A, which has been suggested as a direct link between the dynamic changes of actin cytoskeleton and regulation of gene activity [7]. Our previous study demonstrated that Rho-ROCK signaling, a central regulator of actin cytoskeleton, plays a switch like role in hASC commitment towards osteogenesis or adipogenesis [5]. This prompted us to question whether ROCK downstream target MRTF-A would also have a similar regulatory function in the lineage commitment of human ASCs.

The role of MRTF-A- and SRF-mediated transcription in regulation of differentiation has been previously studied mostly with genomic methods such as gain and loss of function of the MRTF-A gene in rodents [12, 19, 24]. Our approach using MRTF-A inhibition with two pharmacological compounds CCG-1423 [27] and its analog CCG-100602 [28, 30] let us carry on a three-week cell cultures, typical for human MSC *in vitro* differentiation studies, under the influence of the inhibitors. We began by optimizing the functional inhibitor concentrations without cytotoxic effects to hASCs because no prior data was available. Both inhibitors had dose-dependent decreasing effect on cell adhesion and viability, as studied with LIVE/DEAD assay. Based on the CyQUANT analysis of cell proliferation, the inhibitors decreased cell number concentration-dependently, and the cell response was cumulative over time. The inhibitor effect was also dependent on the culture medium.

Previous *in vivo* and *in vitro* studies have suggested that MRTF-A and SRF transcription factors have a negative role in regulation of adipogenesis. This means that MRTF-A activity or overexpression is linked to decreased adipogenesis, whereas knockdown or diminished MRTF-A signaling to enhanced adipogenic differentiation fate [12, 18–20, 24]. As expected, we found that inhibition of MRTF-A activity supported the adipogenesis of hASCs. Based on ORO staining of neutral lipids and the quantitative analysis of lipid droplet area, inhibitor treatment stimulated adipogenic commitment, and large lipid droplets were detected throughout the culture area. MRTF-A inhibition also supported the maturation process, characterized with the enlargement and fusion of individual droplets [16]. In AM control with unaffected MRTF-A activity, the lipid droplets formed scarce, unevenly distributed clusters.

We also studied the adipogenesis with ICC staining of Plin1, which is one of the perilipin family proteins of the phospholipid monolayer shielding the lipid droplet hydrophobic core [13, 44, 45]. Intense staining of Plin1 was found in all AM conditions, although large droplets with Plin1 on the surface were only detected in MRTF-A inhibitor-treated hASCs. It is possible that some lipid droplets were lost during the staining protocols, because mature adipocytes lose their attachment to the culture platform [14]. In basic culture medium, MRTF-A inhibitor enhanced Plin1 production of hASCs. Furthermore, the lipid droplet size was slightly increased, indicating that the molecular intervention was enough to support adipogenesis even without adipogenic culture supplements. Our results are in coherence with previous findings that Plin1 is one of the PPAR*γ* target genes that is upregulated by MRTF-A depletion in mouse preadipocytes [12].

Recent studies have identified the phenotype of the MRTF-A regulated adipocytes, and their identity was reported to equivalent beige (also called brite) adipocytes [17, 19]. These adipocytes have characteristics of both white and brown adipose tissue; they are multilocular- and mitochondria-rich adipocytes involved in energy dissipation and thermogenic activities [46]. McDonald and coworkers found that the circulating levels of leptin were diminished in the MRTF-A knockout mice compared with the wild type littermates [19]. There is also some evidence that the human adipokine leptin is more associated with white adipose tissue than brown phenotype [47]. Our results of gene expression show that *LEP* was strongly expressed in the adipogenic control condition and markedly downregulated with MRTF-A inhibition. Additionally, the organization of the maturating lipid droplets was different from the typical fat vacuole clusters forming under adipogenic supplements. Therefore, the phenotype of hASCs with MRTF-A inhibition could be somewhat beige-like. However, the molecular identity of these cells will remain to be determined.

Next, we asked if the enhanced adipogenic differentiation with MRTF-A inhibition was linked to a reciprocal reduction in the osteogenic potential of hASCs. Our goal was to carefully study whether early osteogenesis, ECM production, and matrix mineralization were regulated by MRTF-A. Unlike adipogenesis, the relation of MRTF-A and osteogenesis has been demonstrated previously only in one study to our knowledge. Bian and coworkers showed *in vivo* and *in vitro* that the bone development and expression of osteogenic markers, respectively, were negatively affected by the loss of MRTF-A function in mice [24]. SRF knockout has been also reported to decrease the activity of early osteogenic markers RUNX2 and ALP [25]. Likewise, based on our results, MRTF-A inhibition significantly reduced the ALP protein activity stimulated by osteogenic culture condition in hASCs, and *RUNX2A* gene expression was hindered with CCG-1423.

Production and secretion of extracellular proteins and mineralization of ECM are an important part of skeletal development, bone remodeling, and homeostasis [48]. Therefore, we stained two constituents of the organic phase of ECM: COL-1, which provides the elasticity and flexibility to bone [48] and OPN, a regulator of matrix remodeling and tissue calcification [49]. Bian and coworkers found that MRTF-A knockout mice had lower protein expression of COL-1 and OPN [24]. As presumed, we discovered that COL-1 and OPN synthesis and secretion were enhanced by hASCs in OM condition. However, our results revealed an interesting phenomenon that these proteins were sequestrated in intracellular space with MRTF-A inhibition. To study the role of MRTF-A in osteogenic maturation, we examined ECM mineral accumulation with qualitative and quantitative AR analyses. Osteogenic media-induced mineralization was significantly and concentration-dependently reduced with both MRTF-A inhibitors. The decreased ECM mineralization has also been previously reported with MRTF-A or SRF knockout murine cells [24, 25]. These results together denote that the activity of MRTF-A is important in the different stages of the osteogenic commitment of hASCs.

Finally, the actin cytoskeleton and synthesis of actin-related proteins were studied to elucidate the role of MRTF-A in regulating the differentiation fate decision of hASCs by mediating the actin dynamics. Dramatic cytoskeletal changes have been reported to occur early in the differentiation process of the fibroblastic and spindle-like mesenchymal precursor cells into mesenchymal lineages to drive the formation of specialized tissues [1, 2, 14, 50]. Nobusue and coworkers proposed that adipogenesis would require disruption of actin stress fibers and subsequent formation of MRTF-A and G-actin complexes [12]. We discovered that MRTF-A inhibition significantly decreased the coherency of actin orientation of hASCs in BM condition, and a similar trend was seen in the intensity of Phalloidin staining representing F-actin. MRTF-A inhibition also reduced the synthesis of *α*-SMA and MLC indicating to SRF-dependent regulation. Importantly, these cytoskeletal changes were related to the observed moderate enhancement of adipogenesis in BM condition. The adipogenic culture supplements and MRTF-A inhibition caused the hASCs to adopt more spread morphology with reduced parallel alignment of the actin filaments. Furthermore, the observed significant reduction in actin polymerization but relatively unaffected cellular protein level of *β*-actin may suggest that MRTF-A inhibition altered the ratio between G-actin and F-actin. Similar inhibitory effect on F-actin was also demonstrated recently in human intestinal myofibroblasts with CCG-100602 [51]. During osteogenesis, the hASC morphology was relatively spindle-like with predominant parallel actin filaments traversing the entire length of the cells. MRTF-A inhibition decreased the F-actin formation, the parallel-alignment of actin filaments, and the expression of SRF-regulated MLC and pMLC in OM condition. When activated, pMLC is involved in the formation of actomyosin complex contributing to the enhanced intracellular tension, linked to osteogenic course [1, 22]. Thus, the inhibitor-mediated changes in the cytoskeletal and protein level were linked to the suppressed osteogenic outcome. These results signify that MRTF-A regulates the differentiation fate of hASCs together with the biochemical cues by coupling the actin dynamics and target gene expression.

## 5. Conclusions

In summary, we have provided evidence that MRTF-A transcription cofactor is an important regulator of the inverse balance between adipogenesis and osteogenesis of hASCs. Our results showing that MRTF-A inhibitors enhance the lipid droplet formation and maturation indicate that MRTF-A is a negative regulator of adipogenesis. Reciprocally, our novel findings of reduced osteogenesis as a response to MRTF-A inhibition highlight the necessity of MRTF-A activity on the osteogenic outcome of hASCs *in vitro*. MRTF-A translates the cytoskeletal changes to gene transcription via SRF and provides an essential temporarily coupled regulatory signaling node in stem cell differentiation. This study adds to the knowledge on the regulation of differentiation lineage commitment in human stem cells and provides further insight into molecular targets for pharmacological intervention to guide the differentiation fate into the desired direction.

## Figures and Tables

**Table 1:** Reagents used in cytochemical staining.

Antibody type	Antibody	Host species	Dilution	Incubation
Primary	Anti-Collagen I (ab90395)^1^	Mouse	1 : 2000	+4°C, overnight
Primary	Anti-Osteopontin (ab8448)^1^	Rabbit	1 : 100	+4°C, overnight
Primary	Anti-Perilipin-1 (ab3526)^1^	Rabbit	1 : 600	+4°C, overnight
Secondary	Anti-mouse IgG Alexa fluor 488 (A11029)^2^	Goat	1 : 500	+4°C, 45 min
Secondary	Anti-rabbit IgG Alexa fluor 488 (A21206)^2^	Donkey	1 : 500	+4°C, 45 min
—	Phalloidin–Tetramethylrhodamine B isothiocyanate (TRITC)^3^	—	1 : 500	+4°C, 45 min
—	DAPI^3^	—	1 : 2000	RT, 5 min

^1^Abcam, Cambridge, United Kingdom. ^2^Thermo Fisher Scientific. ^3^Sigma-Aldrich.

**Table 2:** Primary and secondary antibodies used in immunodetection.

Antibody type	Antibody	Host species	Dilution	Incubation
Primary	Anti-MKL1/MRTF-A (14760S)^1^	Rabbit	1 : 800	+4°C, overnight
Primary	Anti-MLC (3672S)^1^	Rabbit	1 : 500	+4°C, overnight
Primary	Anti-pMLC (3671S)^1^	Rabbit	1 : 500	+4°C, overnight
Primary	anti-*α*-SMA (14968S)^1^	Rabbit	1 : 1000	+4°C, overnight
Primary	Anti-*β*-actin (sc47778)^2^	Mouse	1 : 2000	RT, 1 h
Secondary	Anti-rabbit IgG-HRP (7074S)^1^	Goat	1 : 2000	RT, 1 h
Secondary	Anti-mouse IgG-HRP (sc2005)^2^	Goat	1 : 2000	RT, 1 h

^1^Cell Signaling Technology, Danvers, Massachusetts, USA. ^2^Santa Cruz Biotechnology, Dallas, Texas, USA.

## Data Availability

The data supporting the results of this study is available upon request from the corresponding author.

## Supplementary Materials

Supplementary Materials Table S1: hASC donor information. Table S2: hASC characterization by surface marker expression. Table S3: primer sequences and accession numbers for qRT-PCR. Figure S1: relative gene expression of *AP2*, *LEP*, and *RUNX2A*. Figure S2: negative controls of the immunocytochemical staining. Figure S3: Western blot and immunodetection of MRTF-A, *β*-actin, *α*-SMA, pMLC, and MLC of a replicate donor cell line. Figure S4: representative images of hASC for image-based analysis of actin orientation.
