# Supplementary material for: Myocardin-Related Transcription Factor A (MRTF-A) Regulates the Balance between Adipogenesis and Osteogenesis of Human Adipose Stem Cells
Source: Stem Cells Int. 2020 Sep 22;2020:8853541. doi: 10.1155/2020/8853541 (PMC7527895; doi:10.1155/2020/8853541)
Supplement: Supplementary Materials — Table S1: hASC donor information. Table S2: hASC characterization by surface marker expression. Table S3: primer sequences and accession numbers for qRT-PCR. Figure S1: relative gene expression of AP2, LEP, and RUNX2A. Figure S2: negative controls of the immunocytochemical staining. Figure S3: Western blot and immunodetection of MRTF-A, β-actin, α-SMA, pMLC, and MLC of a replicate donor cell line. Figure S4: representative images of hASC for image-based analysis of actin orientation. [file 8853541.f1.pdf]

Supplementary material

Supplementary Tables

Table S1. hASC donor information

Donor code	Age	Gender	Harvest site
HFSC 1/13	55	Female	subcutaneous fat from the abdomen
HFSC 11/13	40	Female	subcutaneous fat from the femur
HFSC 4/14	43	Female	subcutaneous fat
HFSC 8/15	63	Female	subcutaneous fat from the abdomen
HFSC 9/15	47	Female	subcutaneous fat from the abdomen
HFSC 10/15	60	Female	subcutaneous fat from the abdomen

Table S2. hASC characterization by surface marker expression

Antigen	Surface protein	Surface marker expression (%)	SD (%)	Fluorophore	Manufacturer
**CD11a**	Integrin alpha L (Lymphocyte function-associated antigen 1)	0.9	0.6	allophycocyanin (APC)	R&D Systems Inc. Minneapolis. MN. USA
**CD14**	Lipopolysaccharide receptor	0.8	0.4	phycoerythrin-cyanine (PECy7)	BD Biosciences. Franklin Lakes. NJ. USA
**CD19**	B lymphocyte-lineage differentiation antigen	0.6	0.3	PECy7	BD Biosciences
**CD34**	Hematopoietic progenitor cell antigen 1	32.0	32.9	APC	Immunotools GmbH. Friesoythe. Germany
**CD45**	RO isoform of leucocyte common antigen	1.9	1.2	APC	BD Biosciences
**CD73**	Ecto-5’-nucleotidase	94,9	4,6	phycoerythrin (PE)	BD Biosciences
**CD90**	Thy-1 (T cell surface glycoproteins)	99.1	0.8	APC	BD Biosciences
**CD105**	SH-2. Endoglin	97.1	5.1	PE	R&D Systems Inc.
**HLA-DR**	Major histocompatibility class II antigen (MHC-II)	0.7	0.5	PE	Immunotools GmbH

Table S3. Primer sequences and accession numbers for qRT-PCR

Gene	5'-Sequence-3'	Product Size (bp)	Accession Number
AP2	forward: GGTGGTGGAATGCGTCATG<br>reverse: CAACGTCCCTTGGCTTATGC	71	NM_001442
LEP	forward: ACAATTGTCACCAGGATCAATGAC<br>reverse: TCCAAACCGGTGACTTTCTGT	73	NM_000230
RPLP0	forward: AATCTCCAGGGGCACCATT<br>reverse: CGCTGGCTCCCACTTTGT	70	NM_001002
RUNX2A	forward: CTTCATTCGCCTCACAAACAAC<br>reverse: TCCTCCTGGAGAAAGTTTGCA	62	NM_001024630.3

Abbreviation: bp, base pair.

Supplementary Figures

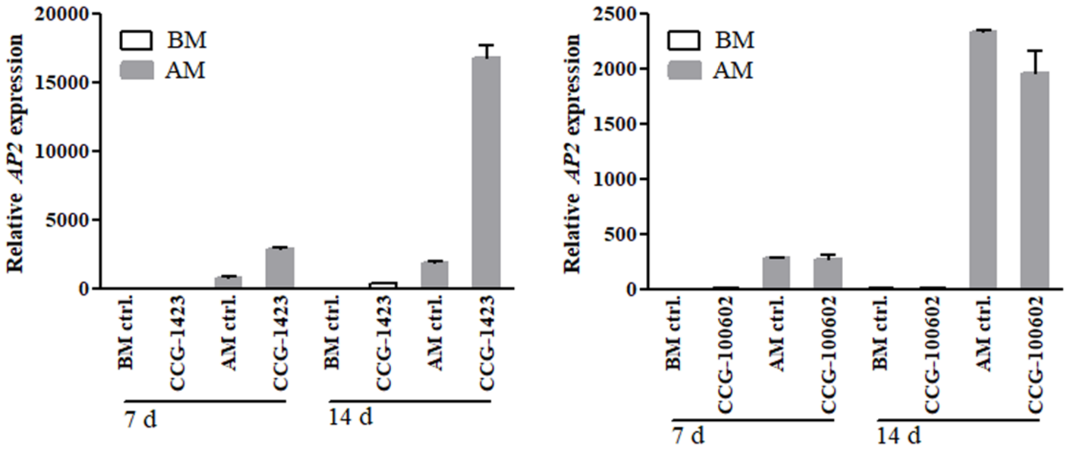

(a)

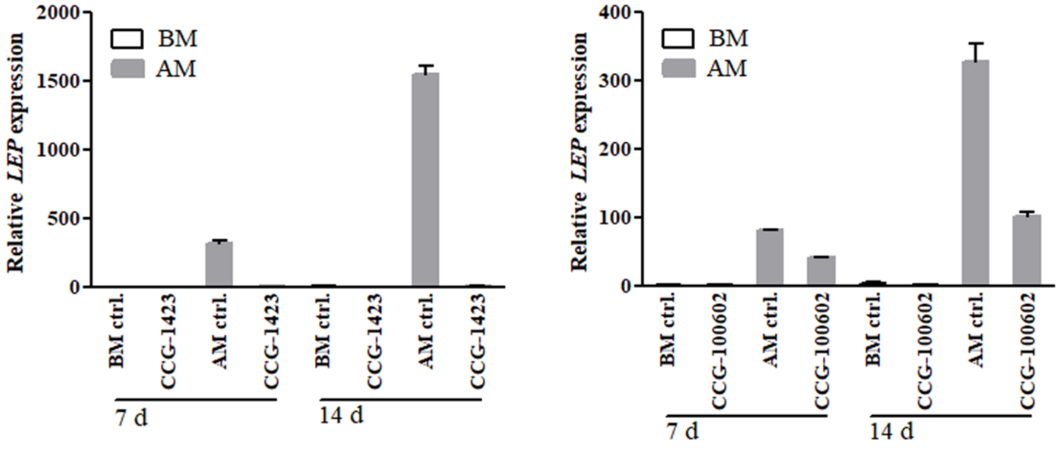

(b)

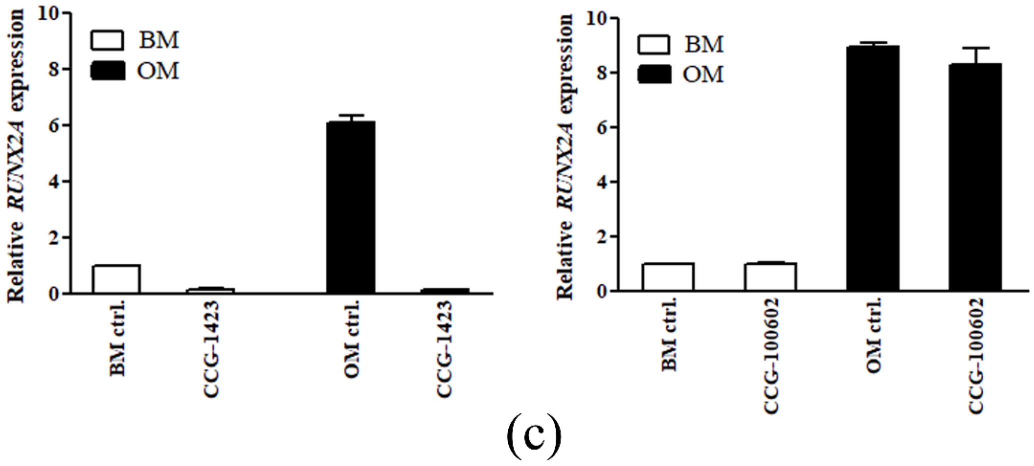

(c)

Figure S1. Relative gene expression of *AP2, LEP,* and *RUNX2A*. The quantitative real-time reverse transcriptase polymerase chain reaction analysis (qRT-PCR) was performed with one donor cell line after 7 and 14 days of culture in BM, OM or AM supplemented with 25 µM CCG-1423 or 12 µM CCG-100602 inhibitors. The hASC were seeded 3160 cells/cm^2 in the CellBIND 6-well plate (Corning). The analysis was performed as described previously (Kyllönen et al., 2013). Briefly, the total RNA was purified from hASC samples, reverse transcribed into cDNA and the qRT-PCR reactions were conducted with AbiPrism 7000 Sequence detection system (reagents from Thermo Fisher Scientific). The relative expressions of adipogenic marker genes (a) *human adipocyte fatty acid-binding protein* (*AP2, also called as FABP4*), (b) human *leptin* (*LEP*) and osteogenic marker gene (c) *human runt-related transcription factor 2a* (*RUNX2A*) at 7d were measured and normalized with the expression of *human acidic ribosomal phosphoprotein P0* (*RPLP0*). N=2, independent biological replicates from 1 donor. Gene sequences and accession numbers are presented in Table S3. Abbreviations: basic medium, BM; osteogenic medium, OM; adipogenic medium, AM.

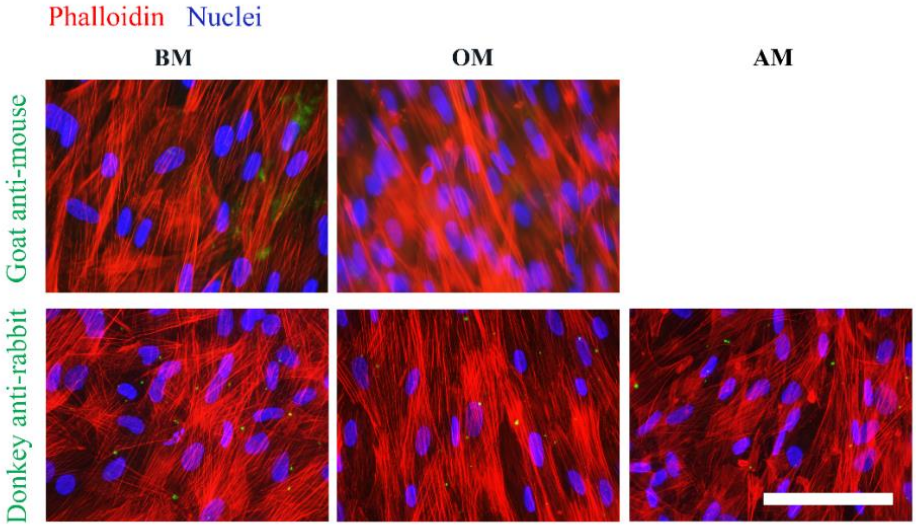

Figure S2. Negative controls of the immunocytochemical staining. The negative control samples of hASC were cultured and treated as the actual samples with the exception that the cells were not treated with primary antibodies. Alexa 488 conjugated secondary antibodies were applied and incubated to confirm that the fluorescence signal is not unspecific, but specifically and selectively binds to the primary antibody and allows detection of the protein of interest. There was an insignificant level of green staining in the secondary antibody controls. Scale bar 100 µm. Abbreviations: basic medium, BM; osteogenic medium, OM; adipogenic medium, AM.

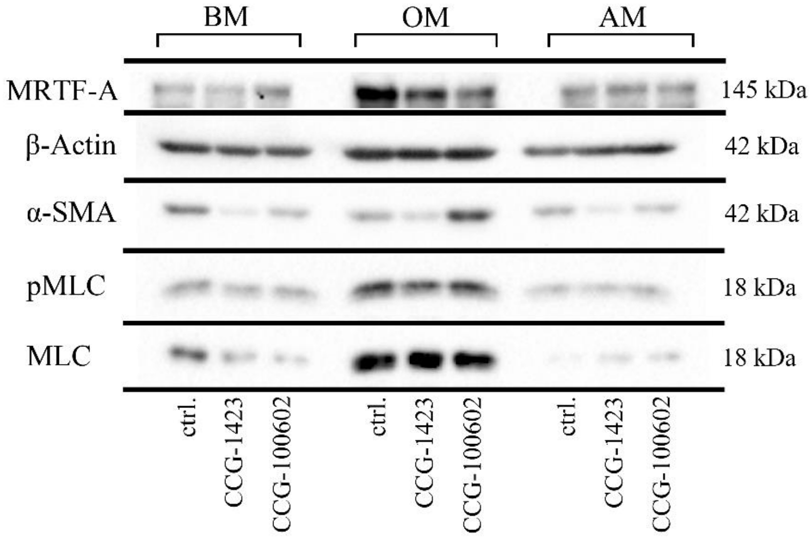

Figure S3. Western Blot and immunodetection of MRTF-A, β-actin, α-SMA, pMLC and MLC of a replicate donor cell line. The hASC were cultured 7 days in BM, OM or AM media supplemented with 20 µM CCG-1423 or 12 µM CCG-100602. Western blot analysis was performed as in the main paper. Abbreviations: basic medium, BM; osteogenic medium, OM; adipogenic medium, AM.

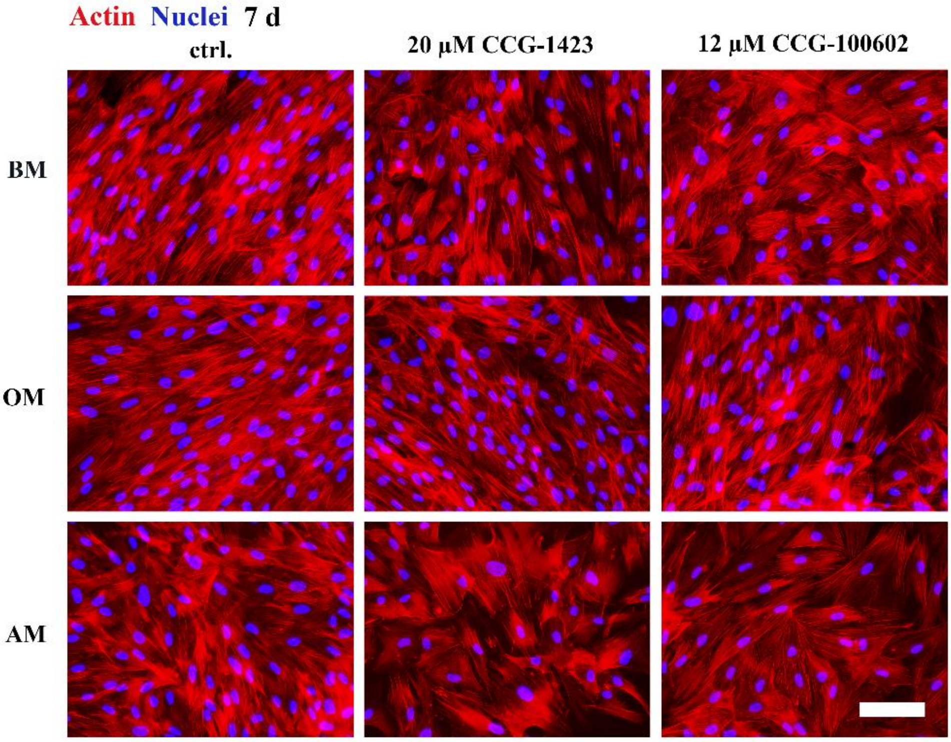

Figure S4. Representative images of hASC for image-based analysis of actin orientation. Actin orientation was analyzed from the images of Phalloidin stained hASC taken with an inverted microscope Olympus IX51, using Alexa 546 filter (red), 20 X magnification and optimally adjusted exposure times for each image to ensure visibility of the cytoskeleton. DAPI stained (blue) nuclei are presented here to indicate the cell number. Scale bar 100 µm.

References

Kyllönen, L., Haimi, S., Mannerstrom, B., Huhtala, H., Rajala, K.M., Skottman, H., Sandor, G.K. and Miettinen, S. (2013). Effects of different serum conditions on osteogenic differentiation of human adipose stem cells in vitro. Stem Cell.Res.Ther. *4*, 17. Published online Feb 15. 10.1186/scrt165 [doi].
